# Rapid drug susceptibility testing of *Mycobacterium tuberculosis* against first-line drugs simultaneously all-in-one plate by using a novel high-sensitive reporter phage combined with the BACTEC MGIT 960 system

**DOI:** 10.1186/s40249-026-01470-5

**Published:** 2026-06-16

**Authors:** Ruiqing Ma, Ying Zhang, Minghua Zhu, Muyang Zhang, Chengcheng Qian, Juan Wu, Zhidong Hu, Mingquan Guo, Xiao-Yong Fan

**Affiliations:** 1 Shanghai Public Health Clinical Center & Shanghai Institute of Infectious Diseases and Biosecurity, Fudan University, Shanghai, 201508 China; 2 Affiliated Hospital of Nantong University, Nantong, Jiangsu Province China

**Keywords:** *Mycobacterium tuberculosis*, Reporter phage, Drug susceptibility testing

## Abstract

**Background:**

Reporter phage assay offers a low-cost and rapid approach for drug susceptibility testing (DST) of *Mycobacterium tuberculosis* (*Mtb*). However, their adoption in clinical laboratories has been limited by challenges such as low detection sensitivity and operational complexity. To address these challenges, we integrated a novel reporter phage into the clinical laboratory workflow for DST of *Mtb*.

**Methods:**

A novel high-sensitive reporter phage, ΦLSN, was constructed by integrating promoter P_*left*_, the ribosome-binding site (RBS) of promoter P_*smyc*_, and nanoluciferase reporter gene (*nluc*) into the genome of the temperature-sensitive phage TM4. Subsequently, a rapid, easy-to-use ΦLSN-based workflow was developed for DST of tuberculosis (TB) specimens following mycobacteria growth indicator tube (MGIT) culture, with the distinct advantage of allowing for simultaneous DST of multiple drugs all-in-one plate within 72 h. The accuracy of the ΦLSN DST assay was evaluated against the traditional solid culture assay, using area under the curve (AUC) analysis based on the DeLong test.

**Results:**

When tested against 62 *Mtb* clinical isolates for susceptibility to first-line anti-tuberculosis drugs (rifampicin, isoniazid, streptomycin, and ethambutol), the ΦLSN DST assay showed sensitivities of 93.8%, 96.4%, 100%, and 80%, and specificities of 100%, 97.1%, 100%, and 100%, respectively. Moreover, preliminary prospective validation against 131 positive MGIT cultures demonstrated that the ΦLSN DST assay achieved sensitivities of 87.5%, 100%, 100%, and 100%, and specificities of 100%, 99.1%, 99.1%, and 99.1% for the four first-line anti-TB drugs within 72 h.

**Conclusion:**

The integration of the BACTEC MGIT 960 with the ΦLSN DST assay provides a high-efficiency and low-cost method for TB DST simultaneously against multiple drugs within 3 days only, presenting potential to enhance the diagnosis and management of drug-resistant TB, especially in the resource-limited regions.

**Graphical Abstract:**

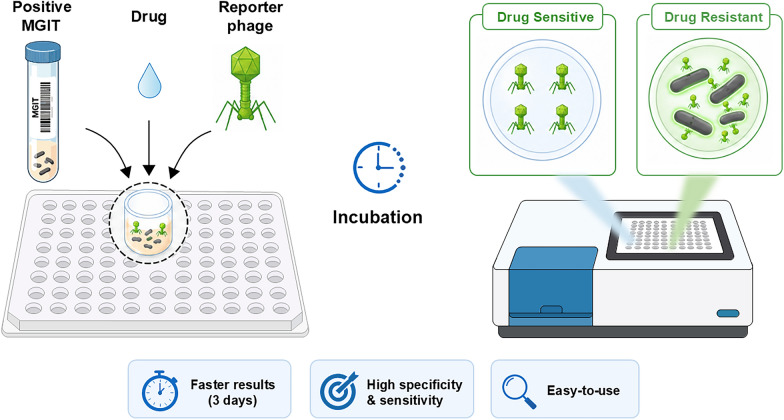

**Supplementary Information:**

The online version contains supplementary material available at 10.1186/s40249-026-01470-5.

## Background

Tuberculosis (TB), caused by *Mycobacterium tuberculosis* (*Mtb*), is a globally prevalent infectious disease primarily transmitted via respiratory route. In 2024, the global burden of TB was estimated at approximately 10.7 million incident cases, of which approximately 390,000 progressed to multidrug-resistant TB (MDR-TB) or rifampicin-resistant TB (RR-TB). However, only 44% of the MDR/RR-TB patients have been diagnosed and treated [1]. Drug-resistant tuberculosis (DR-TB) continues to pose a formidable global public health crisis, especially for economically underdeveloped regions, characterized by limited diagnostic options, substantial mortality and high cost of treatment [2].

Timely resistance detection can increase treatment success and decrease transmission networks for DR-TB. However, slow-growing nature, low bacterial loads in clinical specimens, and considerable genetic variability of *Mtb* collectively hinder laboratory detection efforts [3]. Traditional culture-based drug susceptibility testing (DST) has served as the gold standard for decades. However, the isolation of clinical strains followed by DST via Löwenstein-Jensen (L-J) solid slant is highly time-consuming, requiring at least 6 weeks. The rapid culture-based method for DST involves two rounds of liquid culture by BACTEC mycobacteria growth indicator tube (MGIT) 960 system (Becton, Dickinson and Company, NJ, USA), which can yield results within 3–5 weeks under contamination-free conditions [4]. In recent years, a variety of rapid molecular detection techniques have been developed and endorsed by World Health Organization (WHO), including GeneXpert MTB/RIF [5], Loop-mediated isothermal amplification [6] and gene sequencing [7]. Nevertheless, significant challenges including unresolved resistance mechanisms, numerous potential targets, and substantial expenses limit the applicability of molecular diagnosis to all therapeutic agents [8, 9]. Consequently, current diagnostic tools available in clinical laboratory face a dual constraint: the inherent slowness of culture-based assays, and the high cost and limited drug coverage of molecular assays.

Phage-based method for DST of *Mtb* is considered to be a compromise to solve the constraints, combining with the advantages of high accuracy and low cost of phenotype DST, and the detection rapidity of molecular DST. Over several decades, multiple reporter phages for *Mtb* have been developed by integrating diverse reporter systems into the temperature-sensitive mycobacterial phage TM4. Among these, luciferase reporter mycobacteriophage (LRP) was the first artificially engineered reporter phage, which drive firefly luciferase expression by the *hsp60* promoter [10]. LRP enabled the identification and susceptibility testing of *Mtb* from MGIT cultures, but it exhibited low detection sensitivity and was unreliable for ethambutol (EMB) [11]. *Φ*^2^GFP10, which integrated promoter P_*left*_ and the green fluorescent protein (GFP), enables precise detection of susceptibility to rifampicin (RIF), isoniazid (INH), and streptomycin (STR) for clinical isolates of *Mtb* [12]. Although *Φ*^2^GFP10 was applicable for sputum specimens, it requires sophisticated infrastructure (fluorescence microscopy or flow cytometry) and exhibits low specificity compared with solid culture method [13]. The reporter phages, TM4-nluc [14] and TM4::GeNL [15], utilizing the nanoluciferase (Nluc) and green enhanced nanoluciferase (GeNL) as the reporter cassettes, respectively, exhibit high detection sensitivity to *Mtb* mc^2^6230 and effectively detect susceptibility to RIF and certain second-line anti-TB drugs in-one-plate for clinical isolates. Previously, we constructed a reporter phage with high sensitivity, ΦFN, by combining promoter P_*furAma*_ and *nluc*. An efficient DST workflow for *Mtb* was subsequently established, which demonstrates well performance in detecting susceptibility of clinical isolates to four first-line anti-TB drugs (RIF; INH; STR; EMB) [16]. These nanoluciferase-based reporter phages are capable of identifying low loads of *Mtb* in batch culture, as well as DST to various antibiotics. However, the utility of these phages has so far been reported only for clinical isolates, which require a culture period of 3–8 weeks on solid medium to obtain from clinical specimens.

In this study, we describe the construction of a novel reporter phage with high sensitivity, ΦLSN. By integrating phage-based DST assay with the MGIT 960 system, we then developed an efficient assay for DST of *Mtb* to four first-line anti-TB drugs, which is expected to substantially save detection time and simplify the testing process with high efficiency and low cost of detection in clinical laboratories.

## Methods

### Bacterial strains and culture conditions

All clinical strains were isolated from TB patients at Shanghai Public Health Clinical Center (Shanghai, China) and conventional DST was performed by solid culture on L-J slant in clinical laboratory. *Mycobacterium smegmatis* mc^2^155 (*Msm*) and clinical strains were grown, routinely, at 37 °C in liquid Middlebrook 7H9 broth (Becton, Dickinson and Company, NJ, USA) supplemented with 10% (v/v) oleic acid-albumin-dextrose-catalase enrichment (OADC; Becton, Dickinson and Company, NJ, USA), 0.5% glycerol, and 0.05% Tween 80. Middlebrook 7H11 agar supplemented with 10% OADC, 0.5% glycerol was used for colony-forming units (CFUs) enumeration.

### Expression of reporter cassettes in *M. smegmatis*

The P_*furAma*_-*nluc-Msm* and P_*left*_*-nluc-Msm* strains were obtained as previously described [16]. Promoter P_*left*_ and different ribosome-binding site (RBS) regions were synthesized by Genewiz (Suzhou, China), and *nluc* was provided by Promega (Wisconsin, USA). The *nluc*, RBS and promoter were assembled into pMV306 [17] using the Gibson assembly method [18]. Plasmids were electroporated into *Msm* to achieve integrative expression of the reporter cassettes.

### Construction of phage ΦLSN

The construction of recombinant phage was performed as previously described [16]. In brief, reporter cassette P_*ls3*_*-nluc* was cloned into pYUB854 using the Gibson assembly method. The plasmid was digested with *Pac*I and ligated into the shuttle plasmid phAE159. Bacteriophages were obtained by electroporating the shuttle plasmid into *Msm*.

### Amplification and purification of phage

The amplification of phage was performed as previously described [14]. Briefly, plaques were picked and added to 200 μl MP buffer (50 mmol/L Tris-HCl, 150 mmol/L NaCl, 10 mmol/L MgSO_4_, 2 mmol/L CaCl_2_, pH 7.5). After incubation at 4 °C overnight, phage titer was determined by infection of *Msm*. To obtain high-titer phage stocks, approximately 20,000 plaque-forming units (PFU) were used to infect *Msm*. The phage was layered over a CsCl solution (1.54 g/ml) and centrifuged for 22 h at 96,281 × *g* at 4 °C to reduce the background nanoluciferase protein. The purified ΦLSN phage was used in the further assays.

### Measurement of relative luminescent unit

The Nano-Glo working solution was prepared by diluting the substrate (Promega, USA) 50-fold in Nano-Glo buffer. After mixing with the sample (1:1, vol:vol), the mixture was incubated for 5 min in a flat-bottom 96-well plate. The relative luminescent units (RLUs) were read by a BioTek Synergy H1 plate reader with 125 gain, 0.1 s read time, and 1 mmol/L read height.

### ΦLSN DST assay

For clinical isolates, DST assay was performed as previously described [16]. Briefly, precultured *Mtb*, with an OD_600_ > 0.2, was diluted 1:1000 by Tween 80-free 7H9 media containing different drugs in 96-well plate. 7H9 medium without drug was used as the control. In the wells containing 100 µl of the reaction mixture, the final concentrations of RIF, INH, STR, and EMB were 1 mg/L, 0.1 mg/L, 1 mg/L and 1 mg/L respectively. Wells containing 7H9 and drugs without *Mtb* were used as blanks. After 48 h incubation at 37 °C, ΦLSN was added to a final concentration of 10^7^ PFU/ml and incubated with *Mtb* for 24 h at 37 °C. The RLU was measured as described above, and the remaining luminescence rates (RLRs) value was calculated using the formula: (drug exposure−blank)/(control−blank) × 100%, where “drug exposure” represents the RLU value of a test well with drug exposure; “blank” indicates the RLU value of the well without *Mtb*; and “control” represents the RLU value of the well without drug exposure. The drug-resistance was determined based on the RLR.

For *Mtb* cultured on L-J solid slants, specimens were processed according to the WHO-recommended operational procedure for clinical laboratory [4]. Briefly, *Mtb* were scraped from the solid slants into 7H9 medium without Tween 80 within 1–2 weeks after growth appears. The bacterial mass was vortexed vigorously for approximately 1 min with glass beads to homogenize the colonies. Subsequently, the tubes were left undisturbed for 30 min to let large clumps of bacteria settle. The turbidity of the suspension was adjusted to that of 0.1McFarland number standard. DST assay was performed following the procedures described above for clinical isolates.

For *Mtb* cultured by MGIT 960 system, cultures that tested positive were collected and stored at 37 °C for up to 1 week. Cultures collected on a weekly basis were processed in a single batch. An aliquot of 30 μl (to facilitate the observation of volumetric changes and minimize the risk of sample handling errors) of each culture was added to 70 μl Tween 80-free 7H9 medium with or without drugs in wells. The subsequent DST steps were performed as described above. All above experiments were performed with three technical replicates.

### Statistical analysis

Statistical analyses were performed using GraphPad Prism (GraphPad Software, CA, USA). Two-group comparisons were conducted using Student's *t*-test. Multi-group compararisons were determined by one-way ANOVA with Tukey’s multiple-comparison test. Data are presented as mean ± SD; *P* < 0.05 was considered statistically significant. ROC curves and AUCs comparisons were performed using MedCalc (MedCalc Software, Ostend, Belgium) softwares with Delong test.

## Results

### Construction of a new *M. tuberculosis* reporter phage ΦLSN

**Fig. 1:**
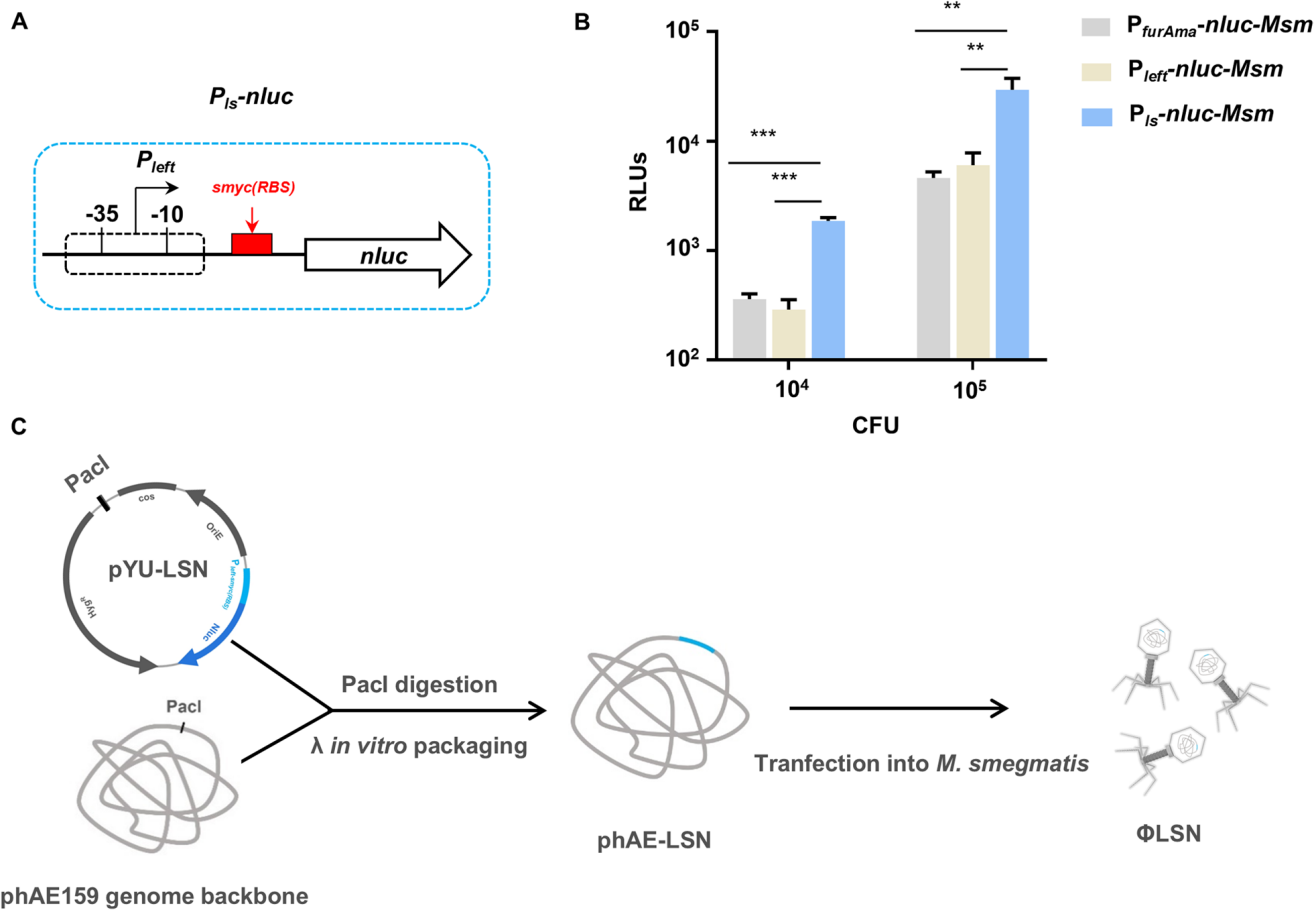
Construction of a reporter phage incorporating a novel reporter cassette P_*ls*_-*nluc*. **A** Schematic diagram of the composition of reporter cassette P_*ls*_-*nluc*. **B** Comparison of the RLUs of P_*ls*_-*nluc* with P_*furAma*_-*nluc* and P_*left*_-*nluc*. The reporter cassettes were integrated into the genome of *M. smegmatis* mc^2^ 155 (*Msm*). Pre-cultured *Msm* carrying distinct reporter cassettes were washed twice with PBS and subjected to tenfold serial dilution. CFUs (X-axis) were determined by spread plate on Middlebrook 7H11 agar. The RLUs (Y-axis) corresponding to different bacterial loads were normalized according to CFUs. Data is presented as mean ± SD; **P < 0.01, ***P < 0.001; one-way ANOVA with Tukey’s multiple comparisons test. **C** Schematic illustration of the construction process of reporter phage ΦLSN. *RLUs* relative luminescent units, *CFUs* colony-forming units

As the RBS of promoter P_*smyc*_ can enhance the gene expression driven by promoter P_*left*_ [19], a new reporter cassette named P_*ls*_*-nluc* was constructed by assembling promoter P_*left*_, RBS of P_*smyc*_ and *nluc* (Fig. 1A). When expressed in *M. smegmatis,* the P_*ls*_*-nluc* reporter cassette produced more relative luminescent units (RLUs) than P_*furAma*_*-nluc* [16] and P_*left*_*-nluc* [14] (Fig. 1B). To optimize the promoter activitiy of P_*smyc*_, three cassettes carrying different RBS-adjacent sequences were constructed and exhibited expression levels comparable to the original cassette P_*ls*_*-nluc* (Fig. S1), and the P_*ls3*_-*nluc* with highest expression of nanoluciferase was subsequently integrated into the genome of temperature-sensitive mycobacteriophage TM4 to generate a new reporter phage ΦLSN (Fig. 1C).

### DST of clinical isolates to four first-line anti-TB drugs using ΦLSN

**Fig. 2:**
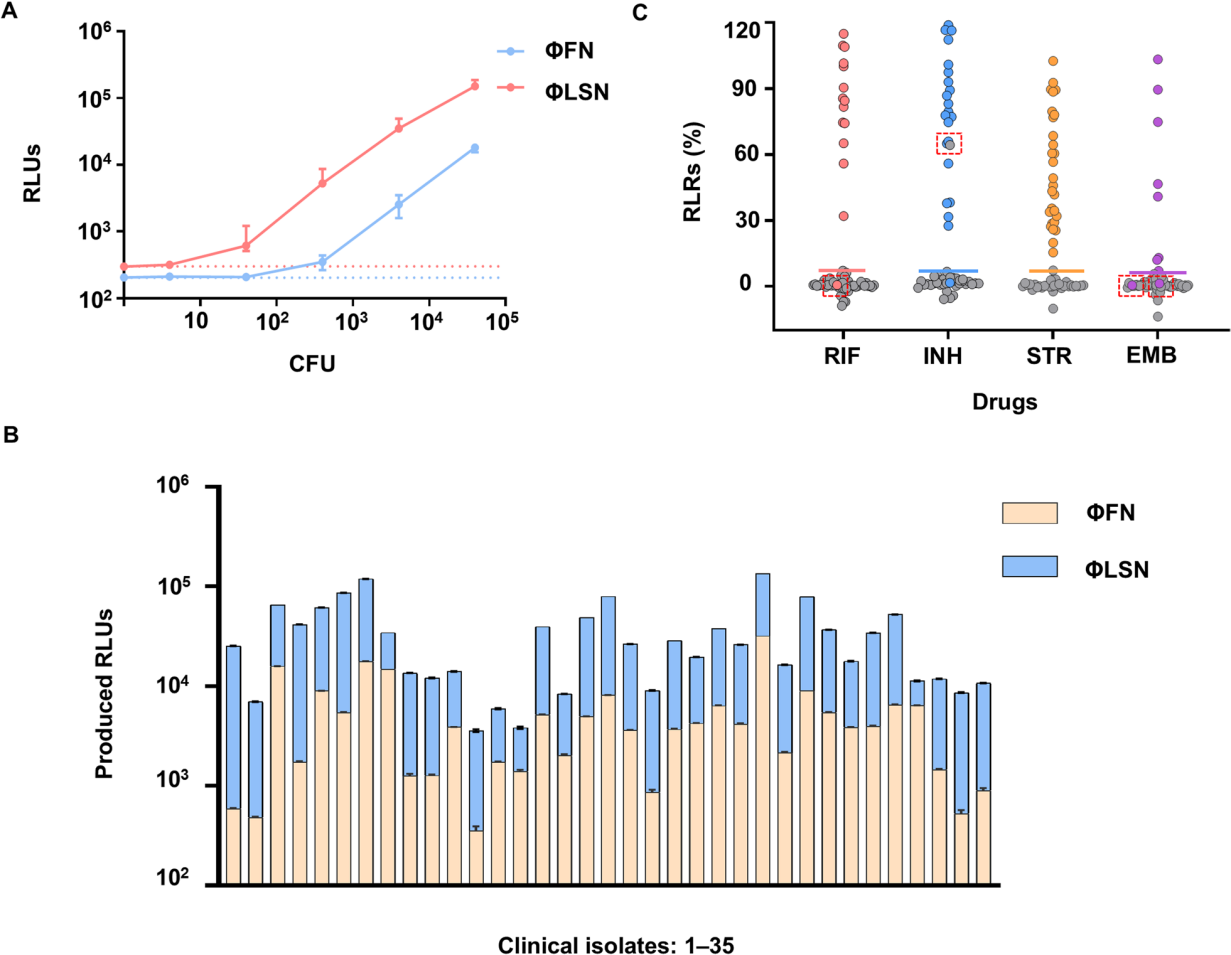
Capability of ΦLSN for DST of clinically isolated *Mtb*. **A** Reporting capability of ΦLSN for *Mtb* isolated from clinical laboratory compared with ΦFN. Ten-fold serial dilutions of *Mtb* gengtype Beijing strain precultured at 37 °C for 48 h were incubated with ΦLSN or ΦFN in 96-well plates for 24 h at 37 °C. The red and blue dashed lines represent the background RLUs of ΦLSN and ΦFN at 10^7^ PFU/ml, respectively. RLUs, relative luminescent units; CFUs, colony-forming units. **B** Produced RLUs of clinical isolates after incubation with ΦLSN. 35 clinical isolates of *Mtb* were detected by ΦLSN and ΦFN following the DST workflow. The produced RLUs were calculated by subtracting the background RLUs value from the measured RLUs value. Each column in the graph represents an individual clinical isolate. **C** The RLRs of 62 clinical isolates assessed by ΦLSN DST assay. Colored and gray dots represent strains resistant to different drugs and non-resistant strains, respectively, as identified by solid culture in clinical laboratory. Colored solid lines represent the RLR cutoffs for different drugs. The red dashed boxes indicate samples with discordant results between the ΦLSN assay and solid culture. *RLRs* remaining luminescence rates, *RIF* rifampicin, *INH* isoniazid, *STR* streptomycin, *EMB* ethambutol

Previously, we established a robust and efficient phage-based DST workflow by ΦFN [16]. Therefore, the reporting capability of ΦLSN was first evaluated by comparing with ΦFN following the workflow. Different bacterial loads of *Mtb* Beijing genotype precultured at 37 °C for 48 h were incubated with ΦFN or ΦLSN for 24 h. The ΦLSN required only a bacterial load of approximately 60 CFUs to produce significant RLUs, in contrast to 600 CFUs for ΦFN (Fig. 2A). Furthermore, when bacterial loads ≥ 100 CFUs, the RLUs produced by the ΦLSN-infected *Mtb* were approximately tenfold higher than that of the ΦFN-infected *Mtb*, which was a trend consistently observed across 35 clinical isolates of *Mtb* with undetermined bacterial loads (Fig. 2B).

To evaluate the accuracy of ΦLSN for DST, a total of 62 clinical isolates of *Mtb* with known resistance to first-line anti-TB drugs were tested by ΦLSN, following the established ΦFN DST assay. All the ΦLSN-infected *Mtb* yielded significant RLUs, and their respective remaining luminescence rates (RLRs) to the different drugs were subsequently calculated (Fig. 2C). In comparison to the outcomes of clinical laboratory, the relative sensitivities of ΦLSN DST assay for RIF, INH, STR, and EMB were 93.8%, 96.4%, 100.0%, and 80.0%, respectively, and the specificities were 100.0%, 97.1%, 100.0%, and 100.0% (Table 1). The areas under the curve (AUC) in the receiver operating characteristic (ROC) curves for RIF, INH, STR, and EMB were 0.965, 0.986, 1.000, and 0.917, respectively (Fig. S2). These findings validate the utility of the reporter phage ΦLSN for DST of clinically isolated *Mtb* against the four first-line anti-TB drugs.

**Table 1:** Performance of ΦLSN in DST to first-line antituberculosis drugs in 62 clinical isolates compared with solid culture

Antibiotic	AUC % (95% *CI*)	RLR cutoff (%)	Sensitivity % (95% *CI*)	Specificity % (95% *CI*)	ΦLSN		Solid culture	
					R^a^	S^b^	R	S
RIF	0.965 (88.4–99.5)	7.2	93.8 (69.8–99.8)	100 (92.3–100.0)	15	47	16	46
INH	0.986 (91.7–100.0)	6.6	96.4 (81.7–99.9)	97.1 (84.7–99.9)	28	34	28	34
STR	1.000 (94.2–100.0)	7.2	100.0 (88.4–100.0)	100.0 (89.1–100.0)	30	32	30	32
EMB	0.917 (81.9–97.2)	5.5	80.0 (44.4–97.5)	100.0 (93.2–100.0)	8	54	10	52

*AUC* area under curve, *RLR* remaining luminescence rate, *RIF* rifampicin, *INH* isoniazid, *STR* streptomycin, *EMB* ethambutol

^a^Resistant

^b^Susceptible

### Establishment of an efficient ΦLSN-based DST assay for cultures of tuberculosis specimens

**Fig. 3:**
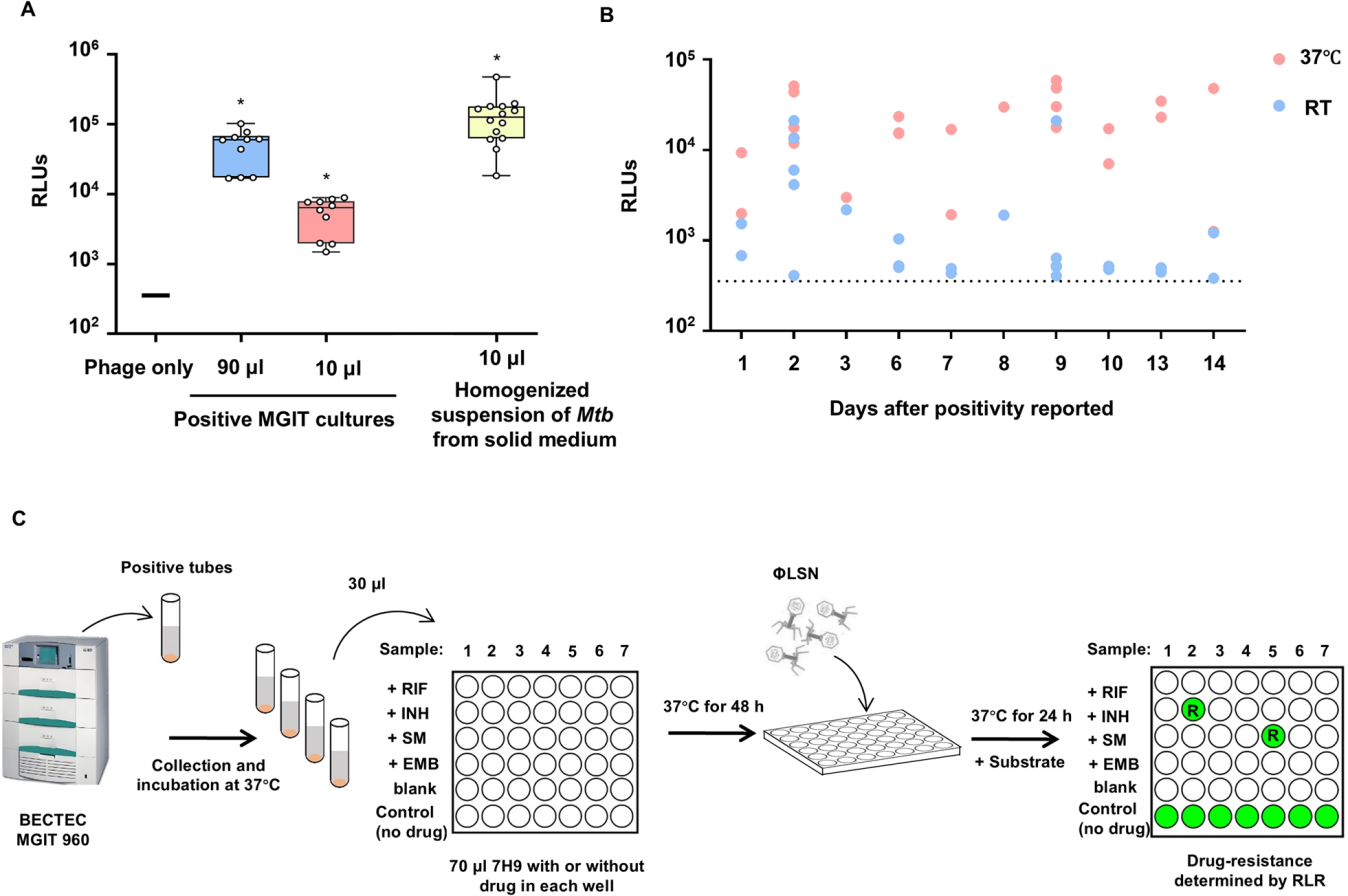
Establishment of an efficient ΦLSN-based DST workflow with positive MGIT cultures. **A** Reporting capability of ΦLSN for *Mtb* cultured by liquid and solid media in clinical laboratory. Different volumes of positive MGIT cultures (time-to-positivity ≤ 24 h) derived from 10 clinical specimens and 10 μl of homogenized supernatants of solid cultures derived from 14 clinical specimens were detected by ΦLSN following the procedure of DST assay. Data is presented as mean ± SD; *P < 0.05 compared with phage only based on Student’s *t*-test. *RLUs* relative luminescent units, *MGIT* mycobacteria growth indicator tube. **B** Recommended storage temperature of positive MGIT culture for ΦLSN-based DST assay. The cultures that tested positive were aliquoted and stored under static conditions at 37 °C and room temperature (RT), respectively. After 2 weeks of collection, all cultures were tested simultaneously using the ΦLSN DST assay. The dashed line represents the background RLUs of ΦLSN. **C** Scheme of ΦLSN-based DST workflow using positive MGIT cultures. *RLRs* remaining luminescence rates, *RIF* rifampicin, *INH* isoniazid, *STR* streptomycin, *EMB* ethambutol

Given that culture-based methods remain the gold standard for TB diagnosis, we evaluated the feasibility of using ΦLSN to directly detect the drug-susceptibility of *Mtb* cultured by liquid and solid media from clinical laboratory. As the culture medium in MGIT is Tween 80-free, it can be used for ΦLSN DST assay without any preprocessing. Different volumes (10 μl and 90 μl) of positive MGIT cultures derived from 10 clinical specimens were incubated with ΦLSN, and significant luminescent signals were produced in all cultures (Fig. 3A). According to the values of RLUs, the concentration of *Mtb* in the positive culture was estimated to be approximately 10^4^–10^5^ CFUs/ml, which is suitable for the established ΦLSN DST workflow. Furthermore, homogenized suspensions of *Mtb* cultuerd on L-J slant also produced significant luminescent signals upon incubation with ΦLSN.

Next, we investigated how long these specimens could be stored at different temperatures while remaining detectable by ΦLSN, aiming to enable centralized testing after daily collection. A total of 25 MGIT cultures that reported positive in 2 weeks were collected and incubated simultaneously with ΦLSN. On the day of reporting positivity, each culture was aliquoted and stored at 25 °C and 37 °C, respectively. All cultures incubated at 37 °C, even for 14 days, produced significant luminescent signals after incubation with ΦLSN (Fig. 3B). Storage at room temperature (RT) for over 24 h significantly attenuated RLUs production, which exhibited a progressive decline with extended storage time. Moreover, cultures failed to produce luminescent signals due to RT storage for 1 week could be reactivated by a subsequent 3-day incubation at 37 °C (Fig. S3), which offers a practical remedy for the failure of immediate storage at 37 °C.

Given that MGIT 960 system is the fastest culture-based assay currently available for TB diagnosis, we accordingly designed a ΦLSN-based DST workflow for the positive MGIT culture, which doesn’t require any preprocessing step. Generally, cultures in MGIT 960 tubes were directly added to the wells containing different drugs and then incubated for 48 h. After subsequent incubation with ΦLSN for 24 h, the RLU is measured upon substrate addition. Finally, the drug susceptibility of *Mtb* is determined by RLR (Fig. 3C). The entire procedure can be completed within minutes and allows for simultaneous processing of multiple samples in a single batch.

### DST of positive MGIT cultures by ΦLSN

**Fig. 4:**
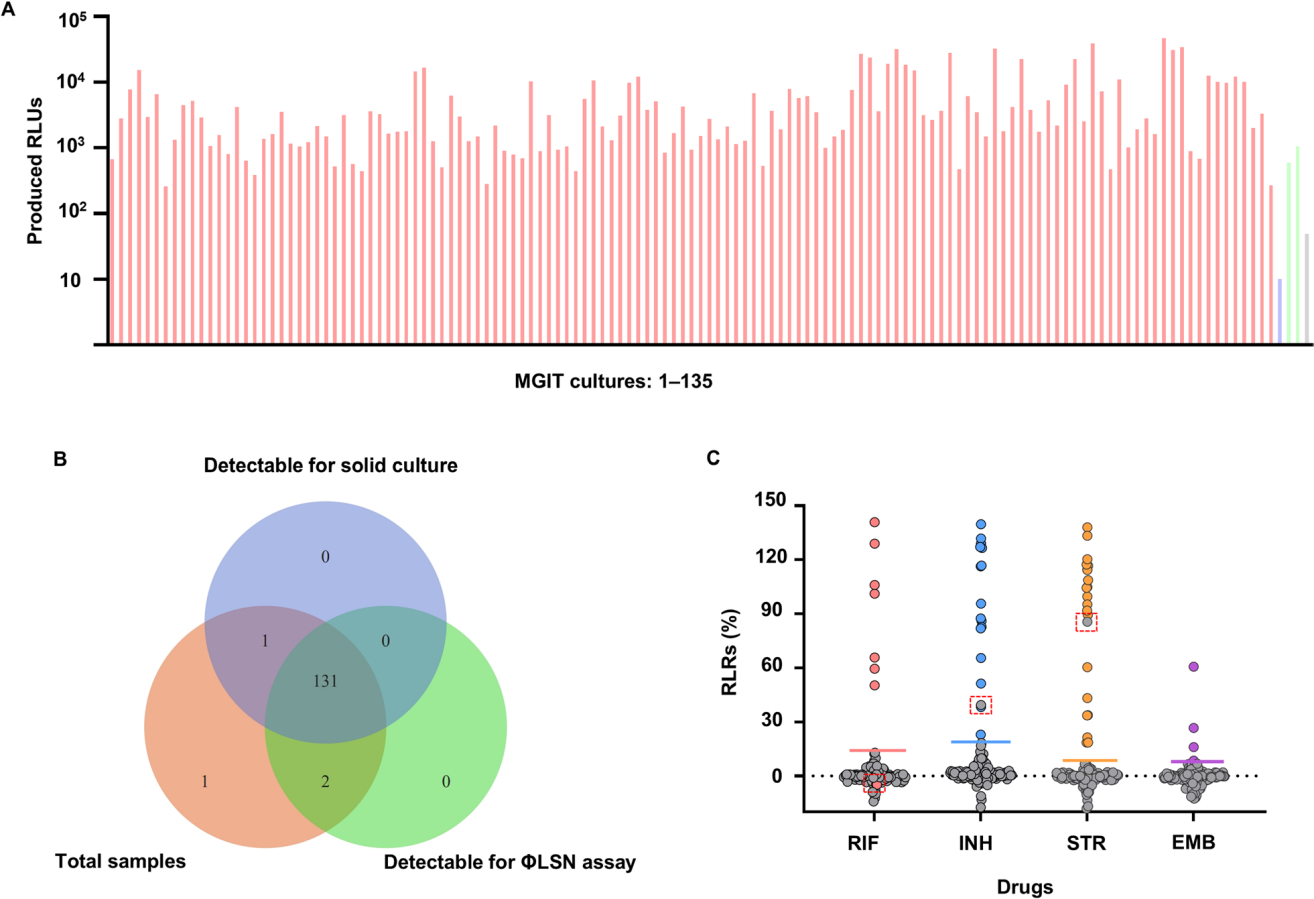
DST of positive MGIT cultures by ΦLSN. **A** Luminescent signals of 135 positive MGIT cultures produced after incubation with ΦLSN. Each column in the graph represents an individual MGIT culture. The columns are categorized by color based on detection methods: red for cultures detectable by both solid culture and ΦLSN; blue for those detectable only by solid culture; green for those detectable only by ΦLSN, and gray for cultures undetectable by both methods. RLUs, relative luminescent units. **B** Venn diagram of the detectability of different methods for the 135 cultures. **C** The remaining luminescence rates (RLRs) of 131 positive MGIT cultures assessed by ΦLSN DST assay. Colored and gray dots represent cultures identified as resistant and susceptible, respectively, by solid culture. Colored solid lines represent the RLR cutoffs for different drugs. The red dashed boxes indicate samples with discordant results between the ΦLSN assay and solid culture. *RIF* rifampicin, *INH* isoniazid, *STR* streptomycin, *EMB* ethambutol

A total of 135 MGIT cultures that reported positivity within 1 week were tested for drug-susceptibility to the four first-line anti-TB drugs using the ΦLSN. Significant luminescent signals were observed in 133 cultures after ΦLSN incubation, while 132 cultures formed colonies on solid medium (Fig. 4A). For the 131 cultures detectable by both methods (Fig. 4B), the RLRs for different drugs were calculated and compared with culture-based assay (Fig. 4C). The relative sensitivity of the ΦLSN DST assay for RIF, INH, STR, and EMB was 87.5%, 100.0%, 100.0%, and 100.0%, and the specificity was 100.0%, 99.1%, 99.1%, and 99.2% (Table 2), respectively. The AUC in the ROC curves for RIF, INH, STR, and EMB were 0.976, 0.999, 0.997, and 1.000, respectively (Fig. S4).

**Table 2:** Performance of ΦLSN in DST to first-line antituberculosis drugs in 131 MGIT 960 culture-positive samples compared with solid culture

Antibiotic	AUC % (95% *CI*)	RLR cutoff (%)	Sensitivity % (95% *CI*)	Specificity % (95% *CI*)	ΦLSN		Solid culture	
					R^a^	S^b^	R	S
RIF	0.976 (93.2–99.5)	12.9	87.5 (47.3–99.7)	100 (97.0–100.0)	7	124	8	123
INH	0.999 (97.1–100.0)	18.4	100.0 (79.4–100.0)	99.1 (95.3–100.0)	17	114	16	115
STR	0.997 (96.6–100.0)	6.6	100.0 (83.9–100.0)	99.1 (95.0–100.0)	22	109	21	110
EMB	1.000 (97.2–100.0)	6.8	100.0 (39.8–100)	100 (97.1–100.0)	4	127	4	127

*AUC* area under curve, *RLR* remaining luminescence rate, *RIF* rifampicin, *INH* isoniazid, *STR* streptomycin, *EMB* ethambutol

^a^Resistant

^b^Susceptible

## Discussion

The high specificity and ease of preparation of bacteriophages for *Mtb* underscore their significant potential for DST applications. In this study, we constructed a novel reporter phage with high sensitivity, ΦLSN, which incorporates an optimized reporter system into the genome of mycobacteriophage TM4 (Fig. 1C). When expressed in *M. smegmatis*, this system achieved a tenfold increase in *nluc* expression compared to the previously reported system based on promoters P_*left*_ or P_*furAma*_ (Fig. 1B), with a comparable enhancement observed in phage-infected *Mtb* (Fig. 2A). To ensure the clinical applicability of ΦLSN for TB diagnosis, all the assessments of reporting capability were performed using *Mtb* clinical isolates, which represents a distinct difference from previously reported highly sensitive reporter phages, such as TM4-nluc [14] and TM4::GeNL [15]. This is critical because bacterial growth rates and expression level of reporter gene vary substantially across different strains. For example, compared to strain *Mtb* mc^2^6320, the clinical isolates produced significantly lower luminescent signals at the same bacterial concentration after incubation with TM4::GeNL. Therefore, we did not define a formal limit of detection (LoD) for ΦLSN in the detection of *Mtb*. Furthermore, the sensitivity of ΦLSN assay in detecting EMB resistance in clinical isolates was lower than that of other drugs (Table 1), potentially due to the high concentration of EMB used. To date, limited research has been focused on the application of phage-based approaches for DST of ethambutol, and an optimal concentration of EMB for such DST assays remain undefined. Thus, evaluating a larger number of EMB-resistant clinical isolates will be necessary to define the optimal concentration.

Our previously reported phage ΦFN demonstrated high accuracy in DST for clinical isolates [16]. Although ΦLSN exhibited superior reporting capability to *Mtb* compared with ΦFN, their sensitivity and specificity for DST showed negligible differences (Table 1). This can be attributed to the bacterial loads (10^4^ – 10^5^ CFUs) used in the assays were sufficient to produce significant luminescent signals. However, ΦLSN is more suitable than ΦFN for the DST of positive MGIT culture because the bacterial loads in wells may fall below 10^3^ CFUs according to the values of RLUs (Fig. 4A).

Cultures derived from both solid and liquid media were tested, and all of them produced observable luminescent signals after incubation with ΦLSN (Fig. 3A). Although we focused primarily on MGIT cultures for DST, developing phage-based DST methods for the more cost-effective solid culture medium is equally valuable, as tuberculosis is most prevalent in economically underdeveloped regions.

We have also considered the potential time delay between positive-MGIT reported and subsequent DST assay, which necessitates the establishment of sample storage conditions. Maintaining the liquid medium at 37 °C after reporting positivity is critical for reliable ΦLSN DST assay, whereas storage at RT significantly impairs the produced luminescent signals (Fig. 3B). If cultures were stored at RT due to unforeseen circumstances, the capability to produce luminescent signals can be rescued by incubation at 37 °C for several days (Fig. S3).

For DST of positive MGIT cultures, we maintained the use of four first-line anti-TB drugs, based on their overall advantages of low cost, minimal side effects, widespread clinical application, and proven therapeutic efficacy. However, workflow for DST of pyrazinamide and various second-line drugs also need to be established. Compared with clinical isolates, the susceptibility of ethambutol (EMB) and the cutoffs of isoniazid (INH) and rifampicin (RIF) show obvious difference (Tables 1 and 2), which potentially due to the limited number of MGIT cultures containing drug-resistant *Mtb*. Therefore, further optimization and multi-center validation are required to establish more reliable parameters and standards for ΦLSN-based DST assay.

According to the clinical laboratory experience, contamination occurs in a subset of positive MGIT cultures, leading to false-positive in the subsequent DST by MGIT 960 assay. To avoid methodological inconsistency**,** solid proportion assay for DST was employed as the reference method instead of the second round of MGIT 960 culture. Among the 135 cultures for DST assays, four outliers were identified: one was invalid for both methods, two failed to grow on solid medium, and one produced weak luminescent signal after incubation with ΦLSN (Fig. 4B). This indicates a low but existent failure rate for both assays, potentially associated with operational factors. Owing to the prospective nature of this study and the low incidence rate of the event, these outliers were excluded from the final detection accuracy analysis**.**

**Fig. 5:**
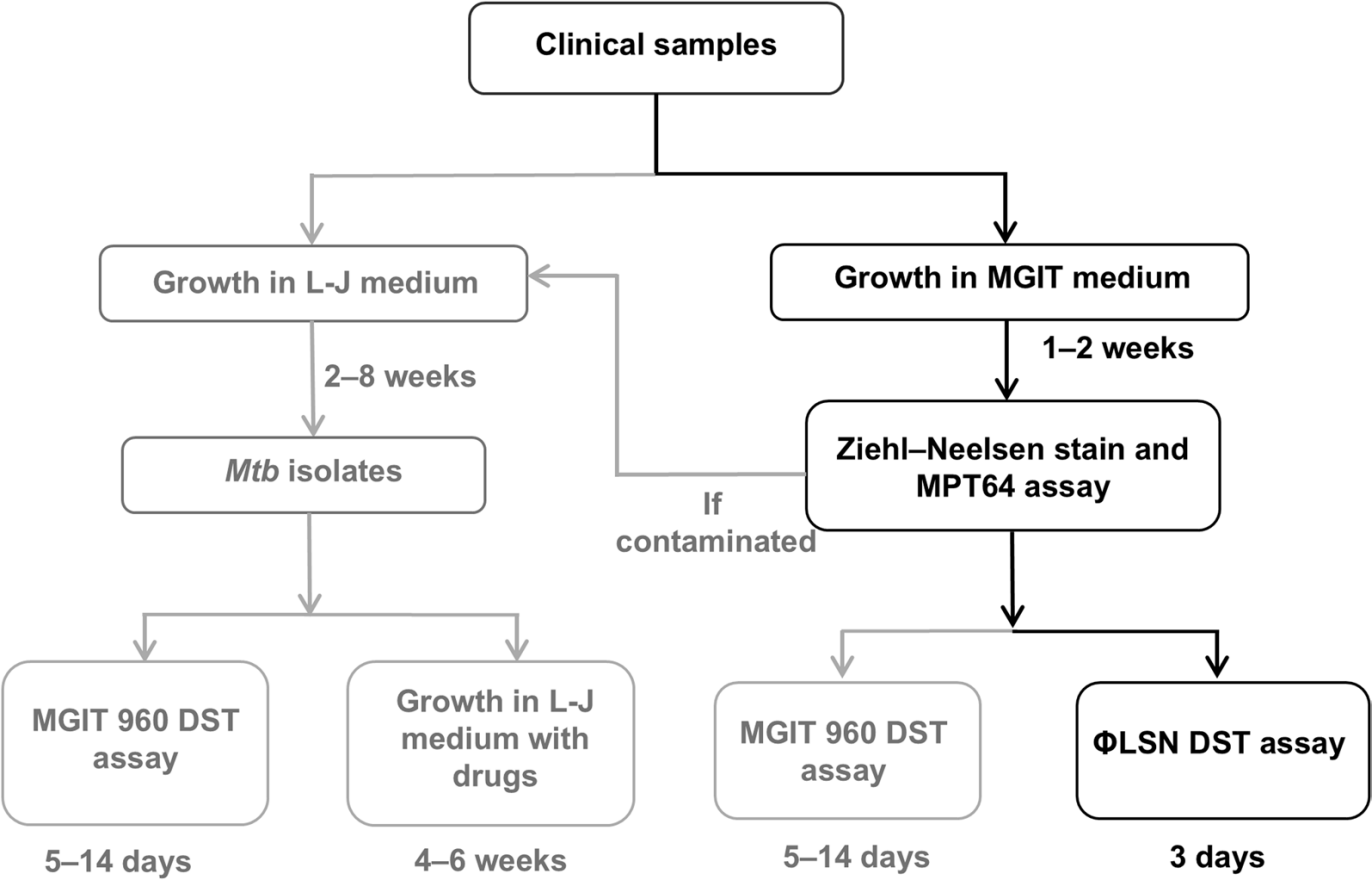
Technical workflow of different culture-based DST methods. Conventional method in clinical laboratories (gray) versus the ΦLSN-based method used in this study (black)

The two-round MGIT 960 culture is the fastest phenotype DST currently available. Although it takes approximately 5–14 days to obtain a result in the second of culturing, 10 days were usually necessary for detecting 95% of the cultures [20]. Thus, the ΦLSN DST assay reduced the testing duration by more than 1 week (Fig. 5) and simplified the workflow by eliminating the dilution step, the aliquot of MGIT 960 positive tube can be added directly for the ΦLSN-based DST assay, which improve the detection efficacy and also save the detection cost in the clinical practice.

## Conclusions

We constructed a novel, high-sensitive mycobacterial reporter phage, and established a robust and rapid DST assay of positive MGIT cultures with low cost and high efficacy to the first-line anti-TB drugs. Preliminary clinical validation of this assay is well-fitted for clinical laboratories, particularly in resource-limited regions, and holds promise as an important tool for addressing the challenges of DR-TB diagnosis and treatment.

## Supplementary Information

Supplementary material 1.

## Data Availability

The datasets used and/or analyzed during the current study are available from the corresponding author upon reasonable request.

## Abbreviations

TB: Tuberculosis
DST: Drug susceptibility testing
*Mtb*: *Mycobacterium tuberculosis*
RBS: Ribosome-binding site
RIF: Rifampicin
INH: Isoniazid
STR: Streptomycin
EMB: Ethambutol
MDR-TB: Multidrug-resistant tuberculosis
RR-TB: Rifampicin-resistant tuberculosis
DR-TB: Drug-resistant tuberculosis
L-J: Löwenstein–Jensen
MGIT: Mycobacteria growth indicator tube
LRP: Luciferase reporter mycobacteriophage
GFP: Green fluorescent protein
RLU: Relative luminescent unit
CFU: Colony-forming unit
RLR: Remaining luminescence rates
AUC: Areas under the curve
RT: Room temperature
NTM: Nontuberculous mycobacteria
*Msm*: *Mycobacterium smegmatis*
OADC: Oleic acid-albumin-dextrose-catalase enrichment
PFU: Plaque-forming units
LOD: Limit of detection
