# Supplementary material for: Rapid drug susceptibility testing of Mycobacterium tuberculosis against first-line drugs simultaneously all-in-one plate by using a novel high-sensitive reporter phage combined with the BACTEC MGIT 960 system
Source: Infect Dis Poverty. 2026 Jun 16;15:69. doi: 10.1186/s40249-026-01470-5 (PMC13270915; doi:10.1186/s40249-026-01470-5)
Supplement: Supplementary file 1 — Supplementary material 1. [file 40249_2026_1470_MOESM1_ESM.pdf]

Supplementary Figures

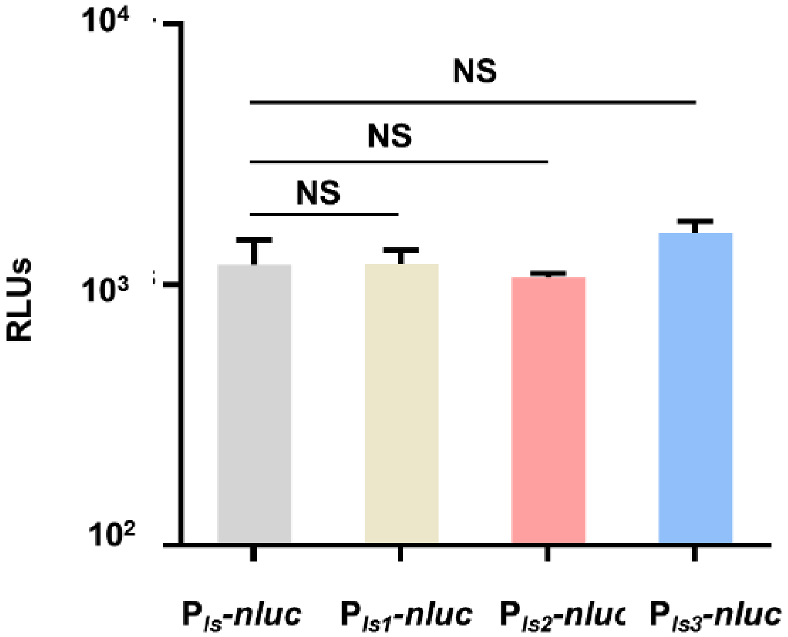

Fig. S1. Determination of expression levels of reporter cassettes harboring different RBS regions in *Mycobacterium smegmatis*. The RLUs (Y-axis) were normalized according to CFUs at 10^4. NS represents no significant difference by one-way ANOVA with Tukey's multiple comparisons test.

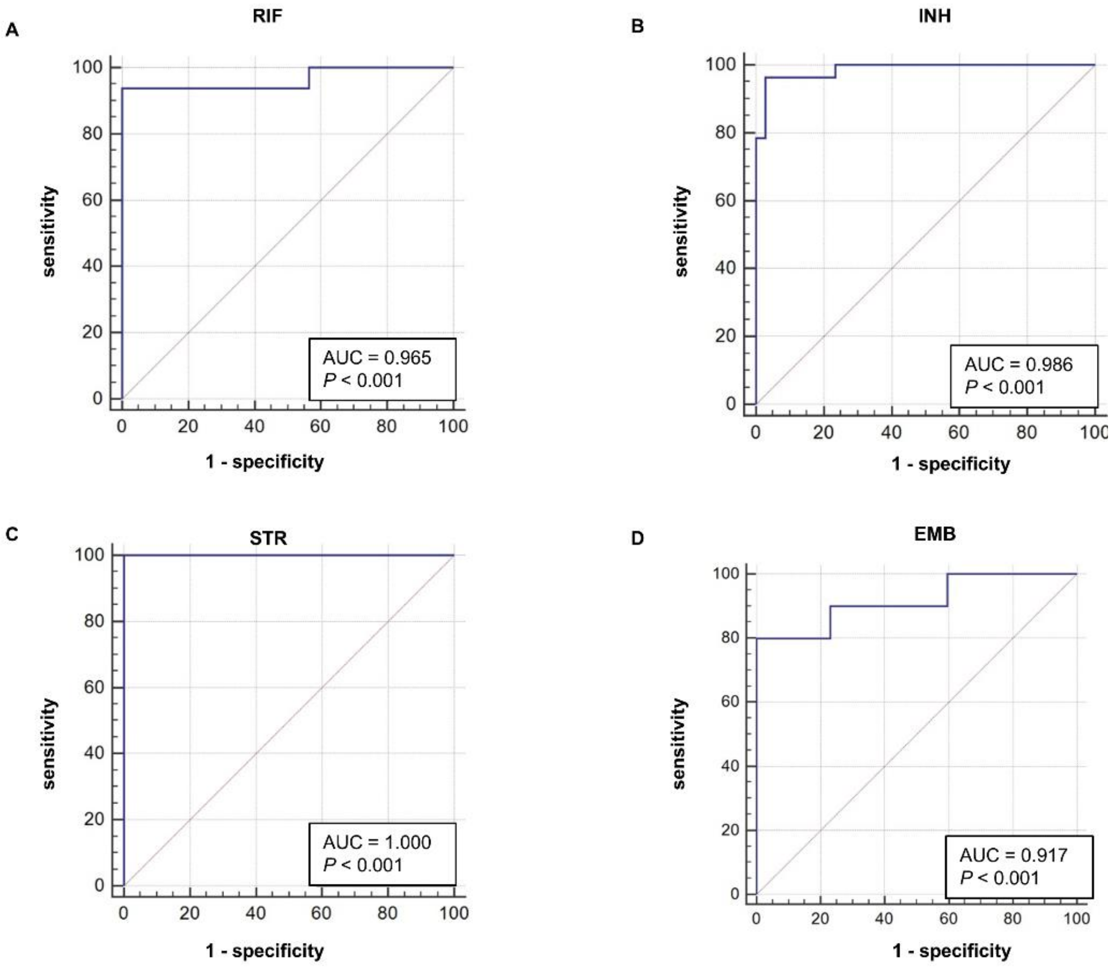

Fig. S2. Receiver operating characteristic (ROC) curves of the ΦLSN DST assay with clinical isolates. The ΦLSN DST assay was compared with solid culture, for the detection of drug-resistance to first-line antitubercular drugs: (A) RIF; (B) INH; (C) STR; and (D) EMB.

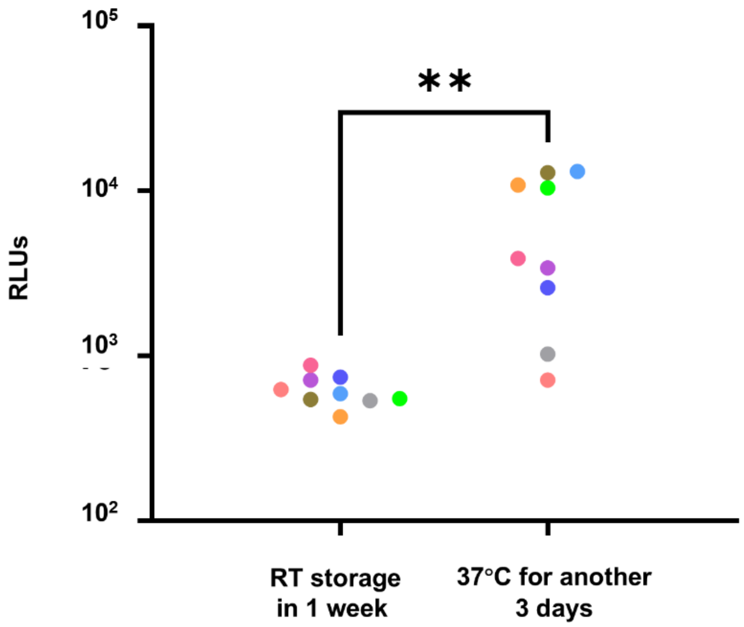

Fig. S3. The remedial effect of 37°C re-incubation on cultures stored at room temperature. Cultures that failed to produce luminescent signals upon ΦLSN infection after one week of storage at RT were collected and re-incubated at 37°C for three days. Each sample is represented by a dot of the same color. **$P < 0.01$; one-way ANOVA with paired *t*-test.

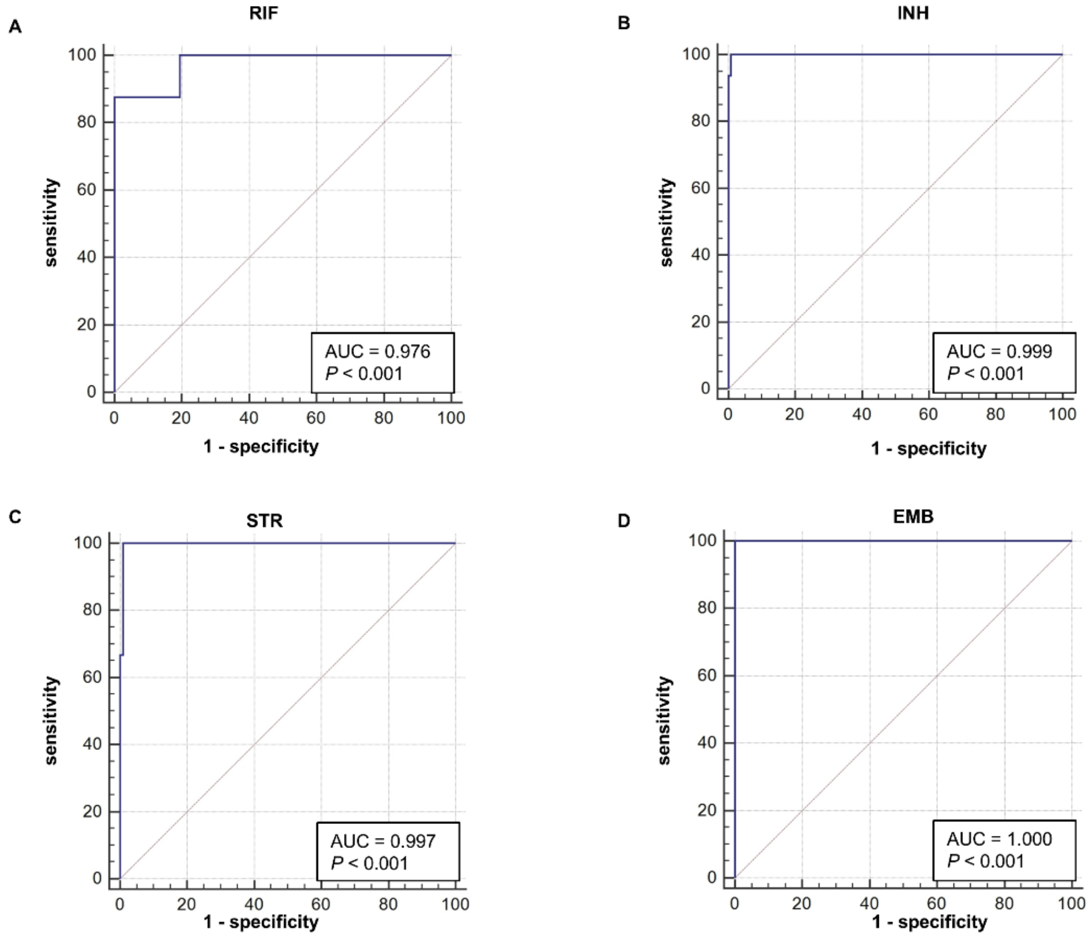

Fig. S4. Receiver operating characteristic (ROC) curves of the ΦLSN DST assay with positive MGIT cultures. The ΦLSN DST assay was compared with the solid culture, for the detection of drug-resistance to first-line antitubercular drugs: (A) RIF; (B) INH; (C) STR; and (D) EMB.
